# Influence of environmental variables in the efficiency of phage therapy in aquaculture

**DOI:** 10.1111/1751-7915.12090

**Published:** 2014-05-20

**Authors:** Yolanda J Silva, Liliana Costa, Carla Pereira, Ângela Cunha, Ricardo Calado, Newton C M Gomes, Adelaide Almeida

**Affiliations:** Departamento de Biologia & CESAM, Universidade de Aveiro, Campus Universitário de SantiagoAveiro, Portugal

## Abstract

Aquaculture facilities worldwide continue to experience significant economic losses because of disease caused by pathogenic bacteria, including multidrug-resistant strains. This scenario drives the search for alternative methods to inactivate pathogenic bacteria. Phage therapy is currently considered as a viable alternative to antibiotics for inactivation of bacterial pathogens in aquaculture systems. While phage therapy appears to represent a useful and flexible tool for microbiological decontamination of aquaculture effluents, the effect of physical and chemical properties of culture waters on the efficiency of this technology has never been reported. The present study aimed to evaluate the effect of physical and chemical properties of aquaculture waters (e.g. pH, temperature, salinity and organic matter content) on the efficiency of phage therapy under controlled experimental conditions in order to provide a basis for the selection of the most suitable protocol for subsequent experiments. A bioluminescent genetically transformed *Escherichia coli* was selected as a model microorganism to monitor real-time phage therapy kinetics through the measurement of bioluminescence, thus avoiding the laborious and time-consuming conventional method of counting colony-forming units (CFU). For all experiments, a bacterial concentration of ≈ 10^5^ CFU ml^−1^ and a phage concentration of ≈ 10^6–8^ plaque forming unit ml^−1^ were used. Phage survival was not significantly affected by the natural variability of pH (6.5–7.4), temperature (10–25°C), salinity (0–30 g NaCl l^−1^) and organic matter concentration of aquaculture waters in a temperate climate. Nonetheless, the efficiency of phage therapy was mostly affected by the variation of salinity and organic matter content. As the effectiveness of phage therapy increases with water salt content, this approach appears to be a suitable choice for marine aquaculture systems. The success of phage therapy may also be enhanced in non-marine systems through the addition of salt, whenever this option is feasible and does not affect the survival of aquatic species being cultured.

## Introduction

Nearly one-third of the world seafood supply comes from aquaculture, the fastest growing food animal-producing sector worldwide (FAO, 2010). Modern aquaculture, however, often suffers from heavy financial losses because of mass mortality caused by bacterial infection, including those promoted by multidrug-resistant bacteria that are easily transmitted through the water and therefore able to infect a great variety of aquaculture species (Almeida *et al*., 2009).

In order to prevent possible diseases in aquaculture, producers often use antibiotics. Nonetheless, this practice is currently discouraged and kept to a minimum in order to avoid the development of drug-resistant bacteria. To overcome the risks of development and spreading of antibiotic-resistant bacteria, it is urgent to develop more environmentally friendly methods to control disease in aquaculture. In line with this idea, the use of phage therapy seems to be a very promising technique, as bacterial diseases are a major problem in the expanding aquaculture industry (Wahli *et al*., 2002; Berthe, 2005; Almeida *et al*., 2009).

Bacteriophages (commonly termed phages) are bacterial viruses that are extremely abundant in nature and are believed to be important in controlling bacterial populations in natural systems (Abedon, 2008), even those known to be multidrug-resistant (Biswas *et al*., 2002; Matsuzaki *et al*., 2003; Wills *et al*., 2005). The use of phages to control bacterial infections in aquatic environments, such as fish diseases, has already been demonstrated in several studies (Nakai *et al*., 1999; Nakai and Park, 2002; Imbeault *et al*., 2006; Vinod *et al*., 2006; Karunasagar *et al*., 2007; Skurnik *et al*., 2007). Phage therapy has also been suggested as a useful approach to control pathogenic bacteria in cultured oyster (Park and Nakai, 2003) and more generally to treat bacterial infections in cultured shellfish (Berthe, 2005), but no reports on bivalve shellfish bacterial control (BC) by phages are currently available. As the host fish and shellfish live in an aqueous media, the therapeutic phage can have a continuous and physiologically close contact with target pathogens in a natural arrangement.

The use of phage therapy requires the understanding of kinetic phenomena unknown in conventional treatments. The kinetic theory indicates that the time of phage application (Nakai and Park, 2002), applied concentration of bacteria and phages, i.e. the multiplicity of infection (MOI) (Cairns *et al*., 2009), and phage survival in culture water of fish farming plants (Nakai *et al*., 1999; Pereira *et al*., 2011), can be critical. It has been shown that a higher protective effect can be promoted by bacteriophages when these are used to control bacteria at an early stage of infection, i.e. phages are simultaneously applied with the bacteria (Nakai and Park, 2002), and an increase in pathogenic bacteria reduction in parallel to that of the MOI (Pasharawipas *et al*., 2011; Prasad *et al*., 2011) or a sooner bacterial reduction at higher MOI (Hsu *et al*., 2000). Pereira and colleagues (2011) showed that the pattern of survival of phages infecting important aquaculture pathogenic bacteria is different in culture waters. An *Aeromonas salmonicida* phage survived in fish-farm water during 90 days. In contrast, the abundance of *Vibrio parahaemolyticus* phage strongly decreased during the incubation period, showing a survival time frame of 16 days (Pereira *et al*., 2011). Nakai and colleagues (1999) showed that anti-*Lactococcus garvieae* phages persisted well in different values of temperature (5–37°C) and salinity (0–70 NaCl g l^−1^) but not in low pH values (3.5, contrary to 6–8).

It has been shown that phage therapy may represent a very useful and flexible tool for the disinfection of aquaculture-farming polluted waters and that time of phage addition, MOI, viral survival and viability are important factors (Almeida *et al*., 2009). However, the effect of physical and chemical properties of aquaculture waters on the efficiency of this approach has never been reported yet. This can be an important aspect to consider, namely if phage therapy would be applied to semi-intensive marine earth ponds in temperate climates, a culture technique widely employed throughout the world, because in these systems, environmental parameters can shift significantly through the year. While Nakai and colleagues (1999) tested the survival of *L. garvieae* phages in various physical and chemical (water temperature, salinity, pH) conditions, they did not study the effect of the various conditions on the efficiency of phage therapy.

The objective of this study was to evaluate the effect of physical and chemical properties of aquaculture waters, such as pH, temperature, salinity and organic matter content, on the efficiency of phage therapy under controlled experimental conditions. The kinetics of phage-bacteria interactions was examined *in vitro* in order to determine the course of the infection and provide a basis for the selection of the most suitable protocol for subsequent experiments in aquaculture systems. A bioluminescent-transformed bacterium *Escherichia coli* (Alves *et al*., 2008) was used as model to follow the phage therapy process in real-time conditions, avoiding the laborious and time-consuming conventional methods of serial dilutions, plating, overnight incubation and counting of colony forming units (CFUs).

## Results

### Phage identification

The phage was identified as a T4-like phage with 95% of homology with the Enterobacteriaceae phage vB_EcoM-VR7 (accession number HM563683). The nucleotide sequence of the phage was deposited in the GenBank database under accession no. KC755108.

### Correlation between bioluminescence and CFUs

**Figure 1:**
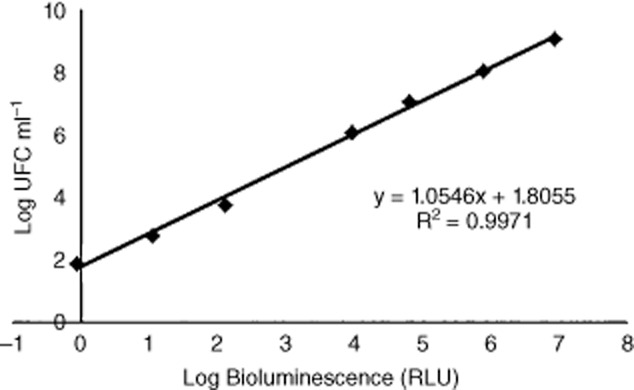
Relationship between the bioluminescence signal and viable counts of an overnight culture of a transformed bioluminescent *E. coli* (∼ 10^9^ CFU ml^−1^) serially diluted in PBS. Bioluminescence is expressed in RLUs and viable counts in CFU ml^−1^. Values represent the mean of three independent experiments; error bars indicate the standard deviation.

It was observed a linear correlation between viable counts and the bioluminescence signal of overnight cultures of the bioluminescent recombinant *E. coli* (Fig. 1).

### Phage therapy assays

#### MOI experiments

**Figure 2:**
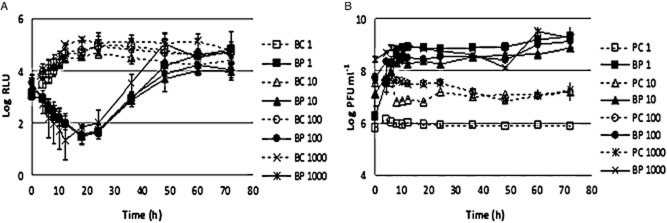
Inactivation of bioluminescent *E. coli* by the phages at different MOI values. (A), bacterial bioluminescence and (B) phage concentration variation along the 72 h experiment. Values represent the mean of three independent experiments; error bars indicate the standard deviation. BC, bacteria control; BP, bacteria plus phage; PC, phage control.

At a MOI of 1, the maximum of bacterium inactivation was 3.3 log, achieved after 24 h of phage therapy. Increasing the MOI to a value of 10 did not significantly increase the rate of inactivation (3 log) after the same period of time [analysis of variance (ANOVA), *P* > 0.05]. At higher values of MOI (100 and 1000), it was shown that the rate of inactivation slightly increases with the MOI, with maximum values of 3.2 log for a MOI of 100 and 3.7 log of reduction at a MOI of 1000, respectively, after 18 and 12 h of phage therapy. After 24 h, the rate of inactivation was about 3 log for both MOI of 100 and 1000 (Fig. 2A). However, this increase in the rate of inactivation with higher MOI values was not statistically significant when compared with the MOI of 1 and 10 (ANOVA, *P* > 0.05). The maximum difference between the different MOI values was of 0.7 log, which corresponds to an increase of 9% in the efficiency of phage therapy (Table 1).

**Table 1:** Maximum reduction obtained after phage therapy of the bioluminescent *E. coli* for each of the tested condition

Parameter	Maximum Reduction		Time Hours
	Log	%	
MOI			
1	3.3	66.3	24
10	3.0	64.1	24
100	3.2	67.6	18
1000	3.7	73.1	12
pH			
6.5	4.6	81.2	24
7.0	3.9	77.7	24
7.4	3.2	67.6	18
Temperature (°C)			
15	4.3	65.0	18
20	4.5	60.0	18
25	3.2	67,6	18
Salinity (g NaCl l^−1^)			
0	3.4	50.0	18
10	4.4	65.9	10
20	5.2	90.0	12
30	5.4	91.6	10
Organic matter			
TSB 1×	3.2	67.6	18
TSB 0.5×	4.2	78.5	24
TSB 2×	5.1	96.6	24
PBS	2.8	46.3	10

Bacterial density in the BCs increased by 1.5 log (ANOVA, *P* < 0.05) during the first 18–20 h of incubation (Fig. 2A). After this time, the bacterial density remained constant until the end of the experiment (ANOVA, *P* > 0.05). The differences in bacterial concentration between different MOI values were not significant (ANOVA, *P* > 0.05).

No decrease of the phage survival was observed during the 72 h of the experiments for the phage alone and for the phage in the presence of its host (Fig. 2B). While the phage control (PC) remained constant during all time (ANOVA, *P* > 0.05), when the phage was incubated in the presence of its host, a significant increase (ANOVA, *P* < 0.05) of 3.4 log was observed for the MOI of 1 (ANOVA, *P* < 0.05). Increasing the MOI to 10, 100 and 1000 revealed a shorter increase on phage survival (about 2 log). Although having similar rates of survival, there is a significant difference between the MOI values of 10 and 1000 (ANOVA, *P* < 0.05), while the others are not statistically different (ANOVA, *P* > 0.05) (Fig. 2B).

#### pH experiments

**Figure 3:**
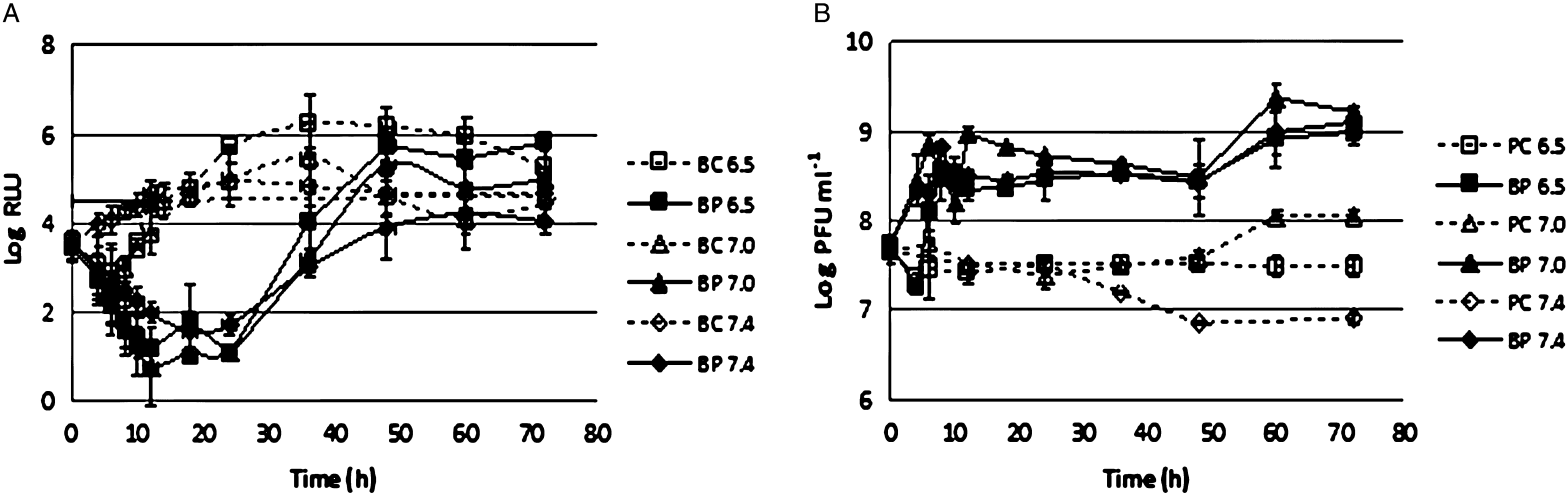
Inactivation of bioluminescent *E. coli* by the phage at different pH values. (A), bacterial bioluminescence and (B) phage concentration variation along the 72 h experiment. Values represent the mean of three independent experiments; error bars indicate the standard deviation. BC, bacteria control; BP, bacteria plus phage; PC, phage control.

When different pH values were tested, it was observed that the rate of inactivation slightly decreased with the increase of the pH; however, the differences among the three values of pH were not statistically significant (ANOVA, *P* > 0.05). At a pH of 6.5, the phage inactivated the bioluminescent *E. coli* by a maximum of 4.6 log after 24 h of incubation. At higher pH values, the maximum rate of inactivation was 3.9 and 3.2 log, achieved after 24 and 18 h of incubation, respectively, at a pH of 7.0 and 7.4. After 24 h, the inactivation was still 3.2 log for the pH of 7.4 (Fig. 3A). The maximum difference between phage inactivation at different pH values was 1.4 log, which corresponds to an increase of 14% in the efficiency of phage therapy (Table 1).

The bacterial density in the BC at a pH of 7.4 increased by 1.4 log (ANOVA, *P* < 0.05) during the first 24 h of incubation, reaching a plateau by this time (Fig. 3A). At pH values of 6.5 and 7.0, the bacterial density decreased by 0.7 log (ANOVA, *P* < 0.05) during the first 8 h of incubation. After this time, the bacterial density increased, after 18 h of incubation, to similar values (ANOVA, *P* > 0.05) to those observed for BC at a pH of 7.4, exceeding even the values of the BC at a pH 7.4 after 24 h (Fig. 3A).

During the time course of the experiments, no decrease on phage survival was observed for the phage in the presence of its host and for the phage alone (Fig. 3B). However, while the PC without bacteria was maintained almost constant throughout the experiment (ANOVA, *P* > 0.05), when the phage was incubated in the presence of its host, an increase of 1.5 log was observed for a pH value of 6.5. The values at a pH of 7.0 increased to 1.2 log, and they were found to be statistically different from those obtained at 6.5 (ANOVA, *P* < 0.05). The highest pH value (7.4) was the one that showed the highest increase in the rate of survival, with values of about 2 log, and it was statistically different only from pH 7.0 (ANOVA, *P* < 0.05) (Fig. 3B).

#### Temperature experiments

**Figure 4:**
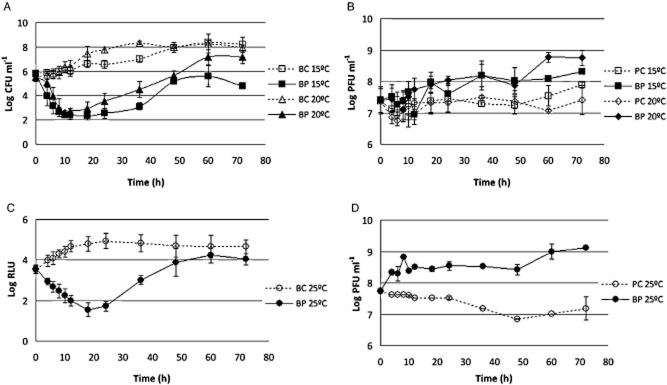
Inactivation of bioluminescent *E. coli* by the phage at different temperature values. (A) bacterial concentration at 15 and 20°C, (B) phage concentration at 15 and 20°C, (C) bacterial bioluminescence at 25°C, and (D) phage concentration variation along the 72 h experiment at 25°C. Values represent the mean of three independent experiments; error bars indicate the standard deviation. BC, bacteria control; BP, bacteria plus phage; PC, phage control.

The rate of *E. coli* inactivation was higher at 15 and 20°C than at 25°C (ANOVA, *P* < 0.05). A maximum decrease of 4.3 log was observed after 18 h when the samples were kept at a temperature of 15°C. When the temperature was increased to 20°C, the rate of maximum inactivation slightly increased to 4.5 log after the same time of incubation. However, the difference between these two temperatures was not statistically significant (ANOVA, *P* > 0.05). At a temperature of 25°C, the maximum rate of inactivation significantly decreased (ANOVA, *P* < 0.05) to 3.2 log after 18 h of incubation (Fig. 4A and C). The maximum difference between phage inactivation at 120 and 25°C was of 1.3 log, which corresponds to an increase of 8% in the efficiency of phage therapy (Table 1).

Bacterial density in the BC incubated at 25°C increased by 1.3 log (ANOVA, *P* < 0.05) during the first 18–24 h of incubation (Fig. 3C). From this moment on, bacterial density remained constant until the end of the experiment (ANOVA, *P* > 0.05). At 15 and 20°C, it also increased during the first 18–20 h of incubation, the increase being higher at 20°C (ANOVA, *P* > 0.05) but reaching similar values (ANOVA, *P* > 0.05) after 36 h of incubation (Fig. 3A).

Phage survival was maintained relatively constant during the time course of the experiments (ANOVA, *P* > 0.05), when the phage was tested alone (Fig. 4B). However, when the phage was incubated in the presence of its host, an increase of 0.4 log was observed when the phage was incubated at 15°C (ANOVA, *P* < 0.05). At higher temperatures (20 and 25°C), the rate of survival increased up to 1.3 log for 20°C and 1.9 log for 25°C. However, the difference between these two temperature values was not statistically significant (ANOVA, *P* > 0.05) (Fig. 4B and D).

#### Salinity experiments

At 0 g NaCl g l^−1^, the maximum rate of inactivation was of 3.2 log after 18 h of incubation. At 10 g NaCl l^−1^, the maximum rate of inactivation was 4.4 log after 10 h of incubation. After 18 h of incubation the rate of inactivation was reduced, although not significantly, to 4 log (ANOVA, *P* > 0.05) but was found to be significantly different from that obtained at 0 NaCl g l^−1^ at the same time (ANOVA, *P* < 0.05). Increasing the salinity to 20 g NaCl l^−1^ also increased the rate of inactivation to a maximum value of 5.2 log after 12 h of incubation. After 18 h, the rate of inactivation was about 4.7 log and was significantly different from that obtained at 10 g NaCl l^−1^ at this time (ANOVA, *P* < 0.05). At 30 g NaCl l^−1^, the maximum inactivation achieved was 5.4 log after 10 h of incubation. After 18 h, the rate of inactivation was reduced to 4.1 log, but that reduction was not statistically significant (ANOVA, *P* > 0.05) and was also not significantly different from that obtained at 20 g NaCl l^−1^ at the same time (ANOVA, *P* > 0.05). The maximum difference between phage inactivation between 0 and 30 g NaCl l^−1^ was of 2.2 log, which corresponds to an increase of 24% in the efficiency of phage therapy (Table 1).

**Figure 5:**
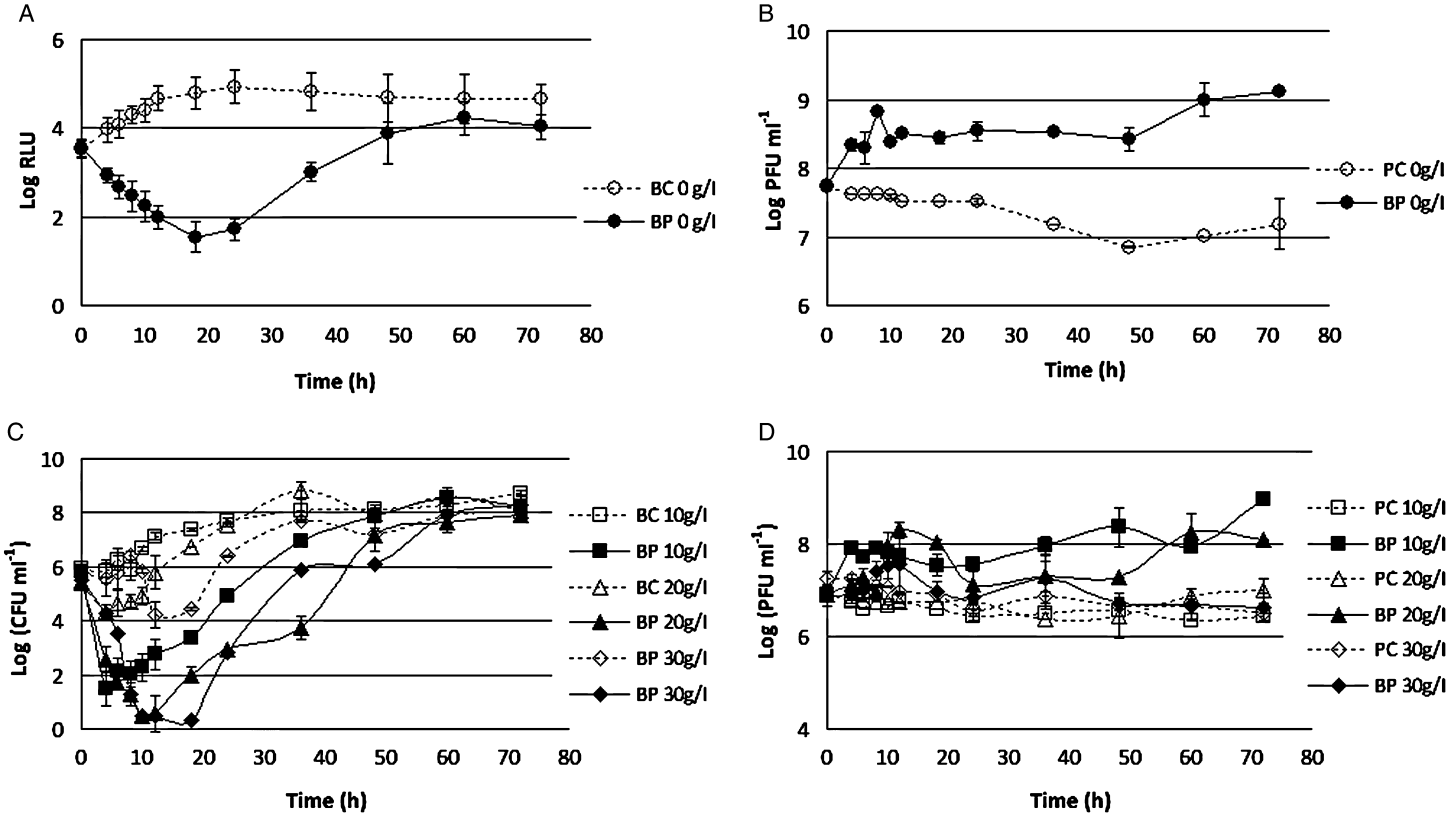
Inactivation of bioluminescent *E. coli* by the phage at different salinity values. (A), bacterial bioluminescence at salinity 0 g l^−1^, (B) phage concentration variation at salinity 0 g l^−1^, (C), bacterial concentration variation at salinities 10, 20 and 30 g l^−1^, and (D) phage concentration variation at salinities 10, 20 and 30 g l^−1^, along the 72 h experiment. Values represent the mean of three independent experiments; error bars indicate the standard deviation. BC, bacteria control; BP, bacteria plus phage; PC, phage control.

Bacterial density in the BC at 0 and 10 g NaCl l^−1^ increased approximately 1 log (ANOVA, *P* < 0.05) during the first 18 h of incubation (Fig. 5A and C). After this time, the bacterial density remained constant until the end of the experiment. In the controls at 20 and 30 g NaCl l^−1^, bacterial density decreased by 0.65 and 1 log, respectively, during the first 4 h (Fig. 5C). The number of bacteria increased after 24 h, reaching values similar to those of BC at 0 and 10 g NaCl l^−1^ (ANOVA, *P* > 0.05).

During the 72 h of the experiments, phage survival was maintained relatively constant when the phage was tested without its host (ANOVA, *P* > 0.05) (Fig. 5B and D). However, when the phage was incubated in the presence of its host, an increase of about 2 log was observed for a salinity value of 0 g l^−1^, which was found to be statistically different from all the other salinity values (ANOVA, *P* < 0.05) (Fig. 5B). At 10 g l^−1^, the increase on phage multiplication was higher (2.5 log) than that obtained at a salinity value of 20 g l^−1^ (about 1 log) and was statistically different from that observed at 30 g l^−1^ (ANOVA, *P* < 0.05) (Fig. 5D).

#### Organic matter experiments

**Figure 6:**
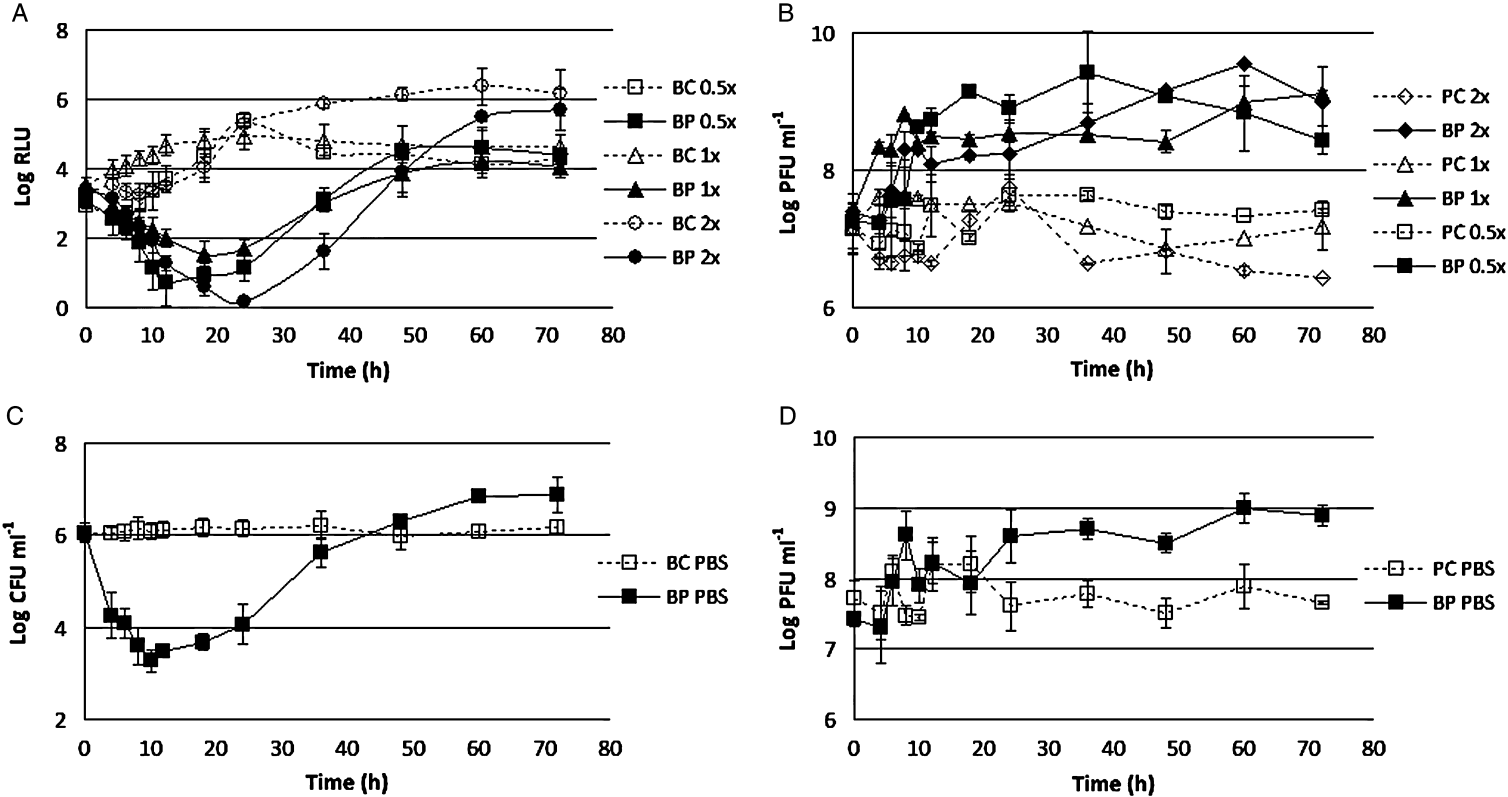
Inactivation of bioluminescent *E. coli* by the phage at different organic matter concentrations. (A), bacterial bioluminescence in TSB, (B) phage concentration variation in TSB, (C) variation of bacterial concentration in PBS and (D) variation of phage concentration in PBS, throughout the 72 h experiment. Values represent the mean of three independent experiments; error bars indicate the standard deviation. BC, bacteria control; BP, bacteria plus phage; PC, phage control.

When the effect of the organic matter concentration was evaluated [Tryptic Soy Broth medium (TSB)0.5×, TSB1×, TSB2×], it was shown that a higher amount of organic matter increases the rate of inactivation. When the assays were done with a normal TSB (TSB1×) growth culture medium concentration, the highest inactivation was 3.2 log and was achieved after 18 h of phage therapy and was not significantly different from that observed after 24 h (ANOVA, *P* > 0.05). When the concentration of TSB was reduced to 50% (TSB0.5×), the maximum rate of inactivation was 4.2 log but occurred later in time (after 24 h of phage therapy) (ANOVA, *P* > 0,05). After 18 h, the rate of inactivation was also not statistically significant from that obtained at TSB1× (ANOVA, *P* > 0.05) and reached a value of 3.4 log. Using TSB with double concentration (TSB2×), the rate of inactivation reached a maximum of 5.1 log after 24 h of phage therapy (ANOVA, *P* < 0.05). After 18 h, however, the difference between TSB with double concentration and regular TSB was not significant (ANOVA, *P* > 0.05), and the rate of inactivation was about 3.4 log (Fig. 6A). When the phage therapy assays were conducted solely in the presence of phosphate buffer saline (PBS) (maximum reduction of the organic matter content), there was still a considerable bacterium reduction (maximum of inactivation 2.8 log) observed after 10 h of phage therapy. After 18 h of treatment, the rate of inactivation was slightly reduced to 2.5 log. When compared with other samples with higher organic matter content, the rate of *E. coli* inactivation recorded for the phage was the lowest one (ANOVA, *P* < 0.05) (Fig. 6C). The maximum difference between phage inactivation under rich nutrient conditions (TSB2×) and poor nutrient condition (PBS) was of 2.8 log, which corresponds to an increase of about 50% in the efficiency of phage therapy (Table 1).

Bacterial density in the BC in TSB1× increased by 1.4 log (ANOVA, *P* < 0.05) during the first 24 h of incubation (Figs. 6A). After this time, the bacterial density remained constant until the end of the experiment (ANOVA, *P* > 0.05). When the TSB was used at 2× and 0.5×, the number of bacteria increased more slowly that when used at 1×, reaching the same values of the BC at 1× (ANOVA, *P* > 0.05) (Figs. 6A). After 24 h of incubation, bacteria in the BC in TSB2× increased by 1 log reaching a plateaux thereafter. Contrarily, after 24 h of incubation, the bacteria in the BC in TSB0.5× decreased by 0.9 log until 36 h, maintained (ANOVA, *P* > 0.05) the bacterial concentration after that (Figs. 6A). The number of bacteria incubated in PBS was constant during the incubation period (Figs. 6A).

No significant decrease of phage survival was observed during the 72 h of the experiment for PCs tested without its host (ANOVA, *P* > 0.05). However, when the phage was incubated in the presence of its host, the rate of phage survival increased for about 2 log after 72 h for the experiments performed on TSB1×. When TSB2× was used, an increase of 2.6 log was observed after 72 h. However, it was found not to be statistically different from the values obtained at TSB1× (ANOVA, *P* > 0.05). Reducing the concentration to 50% (TSB0.5×), or when the assays were performed on PBS, produced only a moderate increase on phage survival of about 1 log, which was found to be not statistically different for both of them (ANOVA, *P* > 0.05) but different from those obtained with TSB1× and TSB2× (ANOVA, *P* < 0.05) (Fig. 6B).

### Prophage detection in the host after phage therapy

No plaques were observed after the addition of mitomycin C to the mixture of bacteria and phages, indicating that lysogeny did not occur.

### Host resistance detection after phage therapy

After incubation in the presence of phages, host bacteria did not show resistance to phage infection. All isolated colonies collected after 120 h of phage application at an MOI of 100 were sensitive to the phage and showed a clear zone of lysis on Tryptic Soy Agar medium (TSA).

## Discussion

Considering that in outdoor facilities aquaculture water is exposed to natural shifts in physical and chemical parameters, thorough investigations addressing different field conditions are required for the design and implementation of effective phage therapy protocols.

The kinetic theory of phage therapy predicts that the MOI could be critical to the bacterial inactivation efficiency. However, there are limited data available on phage effective doses in aquaculture applications. Consequently, before determining the effect of the environmental factors in the efficiency of phage therapy, it is necessary to determine the best MOI to be used. It has been shown, both *in vitro* and *in vivo*, that the reduction of pathogenic bacteria increases in parallel with MOI (Pasharawipas *et al*., 2011; Prasad *et al*., 2011) or that bacterial reduction occurs sooner at higher MOI values (Hsu *et al*., 2000). In treated catfish *Clarias batrachus* serum samples with 4.6 × 10^6^ plaque forming unit (PFU) ml^−1^ and with 9.1 × 10^6^ PFU ml^−1^ of a lytic phage specific to *Flavobacterium columnare*, bacterial abundance was reduced, respectively, by 3 log (58.4%) and 5 log (73.8%) after 96 h, when the animals were challenged with the same bacterial concentration (Prasad *et al*., 2011). A *V. harveyi* phage, isolated from a shrimp pond contaminated with *V. harveyi*, resulted in the inactivation of all *V. harveyi* at an MOI of 20, while an MOI value of 1 resulted in a significant survival of *V. harveyi* (Pasharawipas *et al*., 2011). The addition of specific phages of *Aeromonas hydrophyla*, at a MOI higher than 11.5, to a pure bacterial culture of *A. hydrophyla* reduced the host concentration by three orders of magnitude after 2 h. The addition of the same phage, at a MOI of 0.23, to *A. hydrophyla* challenged pond water of Japanese eel *Anguilla japonica* reduced the host by two orders of magnitude but only after 8 h (Hsu *et al*., 2000). In the present study, the increase in MOI from 1 to 1000 did not promote a significant increase in the efficiency of phage therapy. While the maximum inactivation occurred sooner in time, bacterial inactivation at a MOI of 1000 was not significantly higher when compared with that observed for the other MOI values. As proposed by Nakai (2010) and contrarily to the case of chemicals and other substances, precise initial doses may not be essential in aquaculture because of the self-perpetuating nature of phages revealed by an increasing of phage titres along with bacteria in infected fish or in contaminated waters. As already referred, the number of phages incubated for 12 h in the presence of the host was similar for an initial MOI of 1 and 1000. Moreover, it has been reported that even if the initial number of phages is lower than that of the bacteria, the number of phages after incubation in the presence of their host exceeded that of bacteria after several generations and that the bacterial population are still controlled (Hsu *et al*., 2000; Henriques *et al*., 2013). Accordingly, in this study, an intermediate MOI value of 100 was chosen to determine the effect of environmental factors in the efficiency of phage therapy.

Results show that although phage survival is not significantly affected by the natural variability of environmental parameters in aquaculture waters from temperate climates, the efficiency of phage therapy can be affected by variations on these parameters, namely water salinity and organic matter content.

It has been shown that factors such as temperature, pH and salinity play important roles on the survival of phages (Wu and Chao, 1987; Nakai *et al*., 1999; Pirisi, 2000; Leverentz *et al*., 2001; Cerveny *et al*., 2002; Jepson and March, 2004; Srinivasan *et al*., 2007). However, taking into account that in aquaculture waters, the variation of these parameters is moderate, these physical and chemical parameters have not been described as factors that could significantly affect phage survival in field conditions (Nakai *et al*., 1999). Nothing is known about the effect of organic matter content on phage survival; in this study, phage survival was not significantly affected by the variation of the organic matter content. Phage survival was stable under different conditions of pH, temperature, salinity and organic matter content in the absence of the host during the time course of experimental trials (72 h) and also until at least 5 days (data not shown). However, in the presence of the host, the phage number increased under all tested conditions, even when phages were added at initial low concentrations (i.e. MOI of 1) relatively to the host density.

The pH has been shown to be an important control factor, influencing attachment, infectivity, intracellular replication and multiplication of phages. (Pirisi, 2000; Leverentz *et al*., 2001; 2004; Jepson and March, 2004). Generally, studies on the lytic activity of bacteriophages have shown that pH values less than 5 and over 10 are less efficient and the optimum pH is around a neutral pH of 6–8 (Jarrell *et al*., 1997; Nakai *et al*., 1999; Pirisi, 2000; Jepson and March, 2004). A phage capable of infecting a bacterium attaches to a receptor site on the cell surface using the phage lysozyme to weaken the bacterial cell wall. This process is very important for phage infection. However, pH can interfere with the lysozyme enzyme and/or with other capsids proteins, thus preventing phage attachment to receptor sites on the host cell (Leverentz *et al*., 2001; 2004). Although most phage proteins are stable at a somehow neutral pH, in this study, pH values ranging from 6.5 to 7.4 were tested, as they are included within the optimum neutral range of pH values; phage therapy efficiency was not significantly affected, even at lower pH values, which affected the bacterial growth at the first hours of incubation.

Much like pH, temperature can also interfere with phage attachment, thus preventing phages to infect the host. The stability of the lysozyme increases with lowering the temperature. For instance, mutant phage T4 lysozyme shows maximum stability at 12.5°C (Fennema, 1996), decreasing its stability at temperatures below or above this value because of denaturation. This could explain why the phage of *E. coli*, a T4-like phage, was more efficient under temperatures of 15–20°C than under 25°C, when the bacterial growth was lower.

Bacteriophages have a requirement for salts at low concentrations to be successful during the infection process and growth (Baross *et al*., 1978). At low concentrations, salt ions interact with proteins and stabilize protein structure by neutralizing protein charges. However, at higher concentrations, NaCl increases the thermal denaturation of the proteins and can also adversely affect the structural stability of the phage nucleic acid (Fennema, 1996). In fact, the optimal stable salinity range of a phage of *V. anguillarum* isolated from a fish overwintering pond was 15–45 g l^−1^ (Wu and Chao, 1987). Cerveny and colleagues (2002) showed that two different phages were effective against three different *V. vulnificus* strains with various degrees of virulence, while a third phage that required the presence of seawater to lyse bacteria *in vitro* was ineffective at treating mice in the absence of salt. Silva (2005) showed that a *V. parahaemolyticus* phage was more salt tolerant than two *V. vulnificus* phages. When the *V. vulnificus* bacteriophages were stored at 4°C in salt concentrations of 6–22 and at 30°C in salt concentrations of 6–10 g l^−1^, they had retained activity by day 52. The bacteriophage of *V. parahaemolyticus* remained active in salt concentrations of 6–30 at 4°C and in salt concentrations of 6–22 g l^−1^ at 30°C by day 52. This salt requirement could explain the results obtained in this study, a higher bacterial inactivation in the presence of NaCl. *E. coli* was inactivated more efficiently in the presence of 10, 20 and 30 g NaCl l^−1^ than in the absence of NaCl. Although the presence of 10, 20 and 30 g NaCl l^−1^ delayed the bacterial growth during the first hours of incubation, this delay was not reflected negatively in the phage therapy efficiency.

The host cell must be in an appropriate physiological state to allow productive phage infection. It is well known that bacteria are more susceptible to phage infection in nutrient-rich conditions (Chibani-Chennoufi *et al*., 2004). In the marine environment, as well as in aquaculture systems, bacteria live with considerable nutrients but not as high as in culture media; nonetheless, they continue to replicate phages, maintaining their concentration around one order of magnitude higher than that of themselves (Almeida *et al*., 2001; 2009). In fact, while in the present study, bacteria were inactivated by phages in a poor nutrient medium (PBS), the efficiency of inactivation was lower than that observed when rich nutrient media were used (regular TSB, 0.5× diluted TSB and double concentrated TSB growth medium).

Although phages can effectively reduce the number of viable bacteria by 3–5 logs after 12–24 h of incubation, depending on the experimental conditions, after this period, some bacteria still remained viable and could regrowth, as observed in most of *in vitro* phage therapy studies (Pereira *et al*., 2011; Vieira *et al*., 2012). However, the bacterial population may have decreased to a level low enough to be eliminated by the host immune system, such as fish in the case of aquaculture application. As demonstrated by the mathematical model proposed by Levin and Bull (2004), the combination of phage and host defences has the potential to keep bacterial density below a lethal threshold. In fact, the results of several studies using animal models showed that animal mortality and/or disease symptoms are significantly lower than those observed in challenged non-treated animals and even than those that occur naturally (non-challenged and non-treated animals) (Nakai *et al*., 1999; Hsu *et al*., 2000; Leverentz *et al*., 2001; Park and Nakai, 2003; Wills *et al*., 2005; Srinivasan *et al*., 2007; Prasad *et al*., 2011; Henriques *et al*., 2013). According to the literature, surviving bacteria may be due to the reduced probability of viruses to find host bacteria (Bull *et al*., 2002; Levin and Bull, 2004), a non-replicating condition of surviving bacteria that is physiologically refractory to phage infection (Bull *et al*., 2002), lysogenic conversion (Skurnik and Strauch, 2006) and to the development of phage resistance by the bacterial host (Levin and Bull, 2004).

The hypothesis of bacteria restart growth due to the low probability of an encounter between viruses and host bacteria is not likely, as the increase of MOI of 1–1000 did not increase the efficiency of phage therapy. The hypothesis of non-replicating bacteria to be physiologically refractory to phage infection is also unlikely because following the peak of bacterial inactivation by phages, the remaining bacteria grew at a high rate and reached densities similar to those observed for controls. The occurrence of lysogeny, which can also render the bacterium immune to not only the original phages but also to related phages (Skurnik and Strauch, 2006), could also explain bacterial regrowth. Nonetheless, no evidence of lysogeny was observed during phage therapy experiments. However, it is important to highlight that only a single test (induction by mitomycin C) was used to evaluate the occurrence of lysogenic conversion, and more rigorous testing is needed to exclude this possibility. The results of this study suggest that bacteria that restarted to grow after phage therapy were not resistant to the phage. Similar results have been observed in other studies (Sillankorva *et al*., 2004; Oliveira *et al*., 2009). However, with the approach used in this study, bacteria that restarted growing after phage therapy regained sensitivity to phages only after three streak-plating steps on solid medium. This means that these bacteria were still infected with viruses, which prevented their infection by new phages. More specific tests, such as searching for modifications in bacterial phage receptors, are needed to evaluate the development of resistance and explain the presence of viable bacteria after phage therapy.

It has been suggested that phage-resistant variants of virulent bacteria would not be pathogenic (Anonymous, 1983). Bacterial cell surface components that act as receptors for phage attachment can undergo mutation when bacteria develop resistance to phages. As some of the surface components can also act as virulence factors, the mutated bacteria would not be any more pathogenic. This could explain why viable bacteria remained after phage therapy *in vitro* but do not interfere with the efficiency of phage therapy *in vivo*. Specific *in vitro* and *in vivo* assays, such as evaluation of virulence factors activity before and after phage therapy, are needed in order to confirm this hypothesis.

In a near future, it will be essential to understand how the interaction of environmental factors influences the efficiency of phage therapy in aquaculture systems. The combination of the best conditions of pH, temperature, salinity and organic matter content can improve the efficiency of phage therapy. This can be possible to apply in closed recirculated systems, such as intensive and superintensive fish and shrimp aquaculture, as well as shellfish depuration. Moreover, it can also be used to estimate how the seasonal variation of environmental properties can affect phage therapy efficiency. It would be important, for instance, to forecast how winter rains through the decrease of salinity and increase of nutrient concentration (e.g. because of the run-off of organic matter from the margins of earth ponds) can affect the effectiveness of phage therapy in semi-intensive aquaculture systems with low salinity.

The rapid method for bacterial detection employing a bioluminescent approach was of great importance to study phage/bacterium relationships. With this method, it was possible to evaluate the effect of MOI in phage therapy efficiency, and it was also possible to monitor the phage infection process in real time using a faster, cheaper and much less laborious approach than that of conventional methods.

## Conclusion

It can be concluded that pH, and probably also temperature (15–25°C), is not likely to affect the success of phage therapy in aquaculture conditions. On the contrary, salinity and organic matter content considerably affect the efficiency of the process. While phage inactivation of microorganisms in organic matter clear matrices is not as effective as in rich media, a high inactivation rate was reached, and a better response could be obtained in aquaculture waters where organic matter concentration is considerably higher than in buffer solutions (such as PBS, maximum reduction of the organic matter content tested). As the effectiveness of phage therapy increases in the presence of salt, this approach is an appropriate choice to apply in marine aquaculture systems and possibly its success can be increased in other non-marine aquatic systems if salt can be added to the media.

## Experimental procedures

### Bacterial strain and growth conditions

The genetically transformed bacterium *E. coli* Top10 (Alves *et al*., 2008) was used as a bioluminescent bacteria model in order to follow the phage therapy process in real-time conditions. In experimental systems, a strong correlation between bioluminescence and viable counts can be demonstrated (Marincs, 2000; Rocchetta *et al*., 2001; Alves *et al*., 2008; 2011). Because the optimum light emission conditions for *E. coli in vitro* (25°C, pH 7.4 and 0–5 salinity) (Alves *et al*., 2008) limits the application of this method for the evaluation of the impact of some physical and chemical conditions [low temperature (< 25°C), high pH (> 7.4) and salinity (> 5)] in the efficiency of phage therapy, for these particular conditions, the conventional method of CFUs counting was used.

Fresh plate cultures of transformed *E. coli* were maintained in solid TSA medium (Merck, Darmstadt, Germany) with 100 mg ml^−1^ of ampicillin and 25 mg ml^−1^ of chloramphenicol at 4°C. Before each assay, one isolated colony was aseptically transferred to 10 ml of TSB medium (Merck) previously added with both antibiotics and was grown overnight at 26°C under stirring (120 r.p.m.). Then, an aliquot of this culture (100 μl) was aseptically transferred to 10 ml of fresh TSB medium with antibiotics and grown overnight at 26°C to reach an optical density of 0.700, corresponding to about 10^8^ cells ml^−1^ and to a relative luminescence unit (RLU) of approximately 6 log.

### Correlation between bioluminescence and CFUs

To evaluate the correlation between CFUs and the bioluminescent signal (in RLUs) of the transformed *E. coli* strain, the bacterium was grown under the aforementioned conditions. The fresh overnight bacterial culture was serially diluted (10^−1^ to 10^−7^) in PBS. Non-diluted (10^0^) and diluted aliquots were pour-plated on TSA medium (1 ml) and, simultaneously, were read on a luminometer (1 ml) (TD-20/20 Luminometer, Turner Designs, Inc., Madison, WI, USA) to determine the bioluminescence signal. Three independent experiments were performed, and the results were averaged.

### Phage isolation and purification

A water sample collected from the Corte das Freiras aquaculture of Ria de Aveiro (Aveiro, Portugal, 40°37′54.94′ N & 8°40′9.76′ W) was filtered through a 0.45 μm pore size polycarbonate membranes (Millipore, Bedford, MA, USA). The filtrate was added to double-concentrated TSB medium with 1 ml of a fresh culture of the bioluminescent transformed *E. coli*. The mixture was incubated at room temperature for 18 h at 80 r.p.m. and then filtered through a 0.2 μm membrane (Millipore). Chloroform at a final concentration of 1% was added to the supernatant. The titre of the supernatant was determined by the double-layer agar method using TSA as culture medium. The plates were incubated at 25°C and examined for the presence of lysis plaques after 12 h.

Three successive single-plaque isolations were performed to obtain a pure phage stock. All lysates were centrifuged at 10.000 *g* during 10 min at 4°C to remove intact bacteria or bacterial debris. The phage stock was stored at 4°C and was added of 1% chloroform.

### Phage identification

The extraction of nucleic acid from phage particles was conducted as described by Griffiths *et al*. (2000). Extractions were performed by the addition of 0.5 ml of hexadecyltrimethylammonium bromide extraction buffer and 0.5 ml of phenol-chloroform-isoamyl alcohol (25:24:1; pH 8.0) to each sample. Samples were lysed for 30 s in a FastPrep FP120 (BIO 101/Savant) at a speed of 5.5 ms^−1^, and the aqueous phase containing nucleic acids was separated by centrifugation (16000 *g*) for 5 min at 4°C. The aqueous phase was extracted, and phenol was removed by mixing with an equal volume of chloroform-isoamyl alcohol followed by repeated centrifugation (16000 *g*) for 5 min at 4°C. Total nucleic acids were subsequently precipitated from the extracted aqueous layer with two volumes of 30% (wt/v) polyethelene glycol – 1.6 M NaCl for 2 h at room temperature, followed by centrifugation (18000 *g*) at 4°C for 10 min. Pelleted nucleic acids were washed in ice-cold 70% (v/v) ethanol and air-dried prior to resuspension in 30 ml of Tris-EDTA (TE) buffer [10 mM Tris HCl, 1 mM ethylenediamine tetraacetic acid, pH 8.0]. The DNA extracted was used to amplify the central portion of capsid gene 23 using Mzia 1 (5′-TGTTATIGGTATGGTICGICGTGCTAT-3′) and CAP8 (5′-TGAAGTTACCTTCACCACGACCGG-3′). The reaction was carried in a Multigene Gradient Thermal Cycler from MIDSCI (Valley Park, MO, USA). A reaction mixture of 25 μl was prepared containing 2 × Dream Taq™ PCR master mix (10 × Dream Taq™ buffer, 2 μM dNTP mix and 1.25 U Dream Taq™ polymerase), 4% (v/v) bovine serum albumin (Sigma Chemical, St. Louis, MO, USA), 0.1 μM primers synthesized by IBA (Louvain-la-Neuve, Belgium) and template DNA (*ca*. 10 ng). After 5 min of denaturation at 95°C, 28 thermal cycles of 45 s at 94°C, 45 s at 56°C and 1.3 min at 72°C were carried out. A final extension step at 72°C for 10 min was performed to finish the reaction. PCR products were checked using standard agarose gel electrophoresis (0.8% agarose, 1× Tris-Acetato-EDTA (TAE) buffer; 100 V for 40 min) and red gel staining. PCR products were used as templates in the sequencing reaction that were carried out in an ABI PRISM–BigDye-Terminator v1.1 (Applied Biosystems, Foster City, CA, USA), using reverse primer CAP8.

### Phage therapy assays

The efficiency of bacterial inactivation by phages, for all tested conditions at each time, was determined by comparing the results (CFU ml^−1^ or RLU ml^−1^) obtained in the experiments with the bacterial culture in the presence of phages (test samples) with the results obtained in the BC experiments (bacterial culture without phages) that was incubated in the same conditions of the test samples. In this way, misinterpretation because of the use of two different methods of bacterial quantification and to the use of less favourable growth conditions for the bacterium, such as low pH, temperature and organic matter concentration, and high salinity, was avoided.

Before determining the effect of the environmental factors (physical and chemical) in the efficiency of phage therapy, it was determined the best MOI to be used in the phage therapy experiments. It was tested the MOI of 1, 10, 100 and 1000.

#### MOI selection experiments

In order to obtain 1, 10, 100 and 1000 MOI, 10^5^ CFU ml^−1^ of the overnight bioluminescent *E. coli* culture and a set of serial dilutions of phage suspensions (10^5^ to 10^8^ PFU ml^−1^) were inoculated into 100 ml sterilized glass Erlenmeyers with TSB medium (final volume 50 ml) and incubated at 25°C without agitation (test samples). For each MOI, two control samples were included, the BC and the PC. BC was not inoculated with phages, and the PC was inoculated with the phage but without bacteria. Both controls were incubated exactly as the test samples. Aliquots of test samples and of the bacterial and PCs were collected after 0, 4, 6, 8, 10, 12, 24, 36, 48, 60 and 72 h of incubation for host bioluminescence measurement and phage quantification in the test samples, for host bioluminescence measurement in the BC and for phage quantification in the PC. The host bioluminescence was measured in the luminometer in quadruplicate. The phage titre was determined, in duplicate, by the double-agar layer method after an incubation period of 12 h at 25°C. Three independent experiments were performed for each condition, and the results were averaged.

#### Assessment of the effect of physical and chemical factors

Having into account the annual variability of pH (6.5–7.5), temperature (13–22°C) and salinity (10–35 g l^−1^), the organic matter concentration (0.9–5.6 mg l^−1^) values in the aquaculture systems of Ria de Aveiro, and the optimum light emission conditions for *E. coli in vitro* (25°C, pH 7.4 and 0–5 salinity), the phage therapy assays were designed to independently test each of the mentioned parameters. In the cases (low temperature, high salinity and low organic matter concentration) that were not possible to evaluate the rate of bacterial inactivation by the bioluminescent technique, the bacterial concentration was quantified by the pour-plated method. Aliquots of 1 ml were serially diluted, plated in duplicate and incubated at 25°C for 24 h. Only one MOI (MOI 100) was used to evaluate the effect of the different physical and chemical factors. The general procedure was similar to that described for MOI selection experiments. For each physical and chemical factor studied, three independent experiments were done, and the results were presented as an average mean.

#### pH experiments

In order to evaluate the effect of water pH, overnight cultures of transformed *E. coli* were added to TSB medium with pH values of 6.5, 7.0 and 7.4. During these experiments, the temperature of the samples was kept at 25°C.

#### Temperature experiments

To evaluate the effect of the temperature, the samples were kept at constant temperature (15, 20 and 25°C) in an incubator. The experiments were performed in TSB medium at pH of 7.4.

#### Salinity experiments

To assess the effect of the salinity, TSB medium was added of NaCl with concentrations ranging from 0 to 30 g l^−1^ (0–3% of NaCl). During these experiments, the temperature of the samples was kept at 25°C and the pH at 7.4.

#### Organic matter concentration experiments

To evaluate the effect of the organic matter concentration, phage assays were performed in PBS, diluted TSB medium (0.5×), TSB (1× TSB) and in double-concentrated TSB (2× TSB). The experiments were performed at a pH of 7.4 and a temperature of 25°C.

### Prophage detection in the host bacteria after phage therapy

In order to evaluate if the phage was capable of lysogenic conversion (i.e. has the ability to incorporate its own genome in the bacterial genome), a test using mitomycin C was applied. Samples were collected after 120 h of contact with the host bacteria at an MOI of 100. The samples were stress-induced with mitomycin C (Sigma Chemical) at a final concentration of 1 μg ml^−1^. Cells with temperate phages usually result in the release of the phage (after inducing it by mitomycin C). The samples were incubated overnight at 25°C and centrifuged (10.000 *g*, 10 min) (ThermoHeraeus Pico, Hanau, Germany). The supernatant was checked for the presence of phages by applying the spot test. The absence of a clear zone after stress-inducing indicates that bacteria have no prophages in their genome. Three independent assays were performed.

### Detection of host resistance after phage therapy

A suspension of the phage and of the transformed *E. coli* at an MOI of 100 was incubated at 25°C. After 120 h of incubation, an aliquot was collected and centrifuged at 10.000 *g*, 10 min. The pellet obtained was suspended in TSB medium and subcultured five times in TSB medium at 25°C during 12 h in order to remove the phage particles from the bacteria. To check whether the strain remained sensitive to the phage, the spot test was performed. Three independent assays were done.

### Statistical analysis

Statistical analysis was performed using Statistical Product and Service Solutions (SPSS) (SPSS 20.0 for Windows, SPSS, Inc., Chicago, IL, USA). The existence of significant differences among the different conditions tested for each physical and chemical factor and for the different MOI tested was assessed by one-way ANOVA model with the Bonferroni post-hoc test. For each situation, the significance of differences was evaluated by comparing the results obtained in the test samples after correction with the result obtained for the correspondent control samples (difference between the concentration obtained in the control and the concentration obtained in the test sample) for the different times of each of the three independent assays. Normal distributions were assessed by Kolmogorov–Smirnov test and the homogeneity of variances was assessed by Levene test. A value of *P* < 0.05 was considered statistically significant.
